# The association between features of epicardial adipose tissue and the risks of early recurrence after catheter ablation in patients with atrial fibrillation

**DOI:** 10.3389/fcvm.2025.1480473

**Published:** 2025-02-18

**Authors:** Leiyu Feng, Liming Li, Linpeng Bai, Li Tang, Yintao Zhao, Xiaoyan Zhao

**Affiliations:** ^1^ Henan Key Laboratory of Hereditary Cardiovascular Diseases, Department of Cardiology, Cardiovascular Center, First Affiliated Hospital of Zhengzhou University, Zhengzhou, China; ^2^ Department of Radiology, First Affiliated Hospital of Zhengzhou University, Zhengzhou, China

**Keywords:** atrial fibrillation recurrence, epicardial adipose tissue, atrial fibrillation ablation, computed tomography, attenuation ranges

## Abstract

**Background:**

Epicardial adipose tissue (EAT) remodeling is associated with atrial fibrillation (AF). However, there is limited research on the contribution of EAT to the risk of AF recurrence (AFR). The purpose of this research was to assess the relationship between the risk of AFR after radiofrequency catheter ablation and the volume and attenuation of the EAT.

**Methods:**

We included a total of 123 consecutive individuals who received AF ablation, 31 of whom suffered AFR. The volume and mean density of the whole-heart and periatrial EAT were measured on computed tomography images using four attenuation ranges. The clinical, atrial, and EAT characteristics of patients with and without AFR were compared. Logistic regression was used to identify independent risk factors and to build a model to predict recurrence. The relationship between EAT characteristics and recurrence was analyzed for the subtypes of AF.

**Results:**

The AFR group had a larger left atrial anteroposterior diameter (47.4 ± 7.4 vs. 43.7 ± 8.0 mm), left–right diameter (78.6 ± 7.9 vs. 74.7 ± 9.1 mm), and volume (145.9 vs. 127.0 mL) than the non-recurrence group (*P* = 0.021, 0.037, 0.015, respectively). The total EAT volume in the AFR group was significantly larger than that in the non-recurrence group, for both the overall and persistent AF groups (all *P* < 0.1). The periatrial EAT volume of the AFR group was significantly larger than that of the non-recurrence group for those with persistent AF (*P* = 0.047, 0.048, 0.048, 0.031 for four attenuation ranges). The total EAT volume and left atrial anteroposterior diameter were independent risk factors for AFR (*P* = 0.035, 0.045, respectively).

**Conclusion:**

The EAT volume and left atrial anteroposterior diameter were of great significance in predicting AFR.

## 1 Introduction

Epicardial adipose tissue (EAT) is the visceral fat of cardiac tissue in proximity to cardiac structures and shares blood supply with the cardiac microcirculation. EAT serves as a substantial local reservoir of molecules that can affect the myocardium (1, 2). The growing advancement and accessibility of non-invasive imaging methods such as CT and cardiac magnetic resonance imaging have provided several pieces of evidence on the association between EAT and the risk of atrial fibrillation (AF) (3).

The Framingham Heart Study found that there was a significant correlation between the volume of pericardial fat and the occurrence of AF, even after taking into account the individual's body mass index (4). The growing interest of scientists and clinicians has been sparked by the regional distribution and functions of EAT.

The greatest obstacle to radiofrequency ablation in patients with AF is the unresolved risk of AF recurrence (AFR). After early research identified the pulmonary vein triggers that cause AF, catheter ablation to isolate the pulmonary veins was recognized as an effective therapy for AF (5). Improved approaches such as contact force-sensing catheters, ablation index-guided procedures, high-power short-duration ablation, pulse-field ablation, and pulmonary vein isolation (PVI) are now more effective and durable. However, even with substantial substrate modifications, atrial arrhythmias often recur, particularly in patients with persistent AF (PeAF) (6). Several risk factors for AFR following catheter ablation have been found, however, there is no single indicator that can effectively predict AFR (7).

An increasing number of patients with AF undergo ablation treatments, which often include preprocedural imaging. This enables the investigation of the correlation between EAT and recurrent AF. Several researchers have demonstrated a similar link between increased EAT and postablation AF. Nevertheless, not all the data are consistent (8–10). In previous studies, four attenuation ranges were used to define the heart fat tissue. Thus, the aim of this study was to investigate the effects of total and periatrial EAT CT characteristics based on four attenuation ranges on clinical outcomes in patients with AF undergoing catheter ablation.

## 2 Methods

This retrospective study was approved by the Institutional Review Board (2023-KY-1286), and the requirement for informed consent was waived.

### 2.1 Study population

A total of 362 consecutive adult patients diagnosed with AF who received radiofrequency catheter ablation (RFCA) at the First Affiliated Hospital of Zhengzhou University from January 2020 to January 2021 were screened for inclusion. Ultimately, 123 patients met our inclusion criteria. The inclusion criteria were as follows: initial RFCA was performed in patients with AF, CT imaging was performed before RFCA, and complete follow-up data were collected. Patients who had the following conditions were excluded: rheumatic valvular heart disease, congenital heart disease, pericardial effusion, or infectious endocarditis. Patients for whom the CT images contained artifacts that affected parameter measurements were also excluded.

### 2.2 Data collection

**Figure 1:**
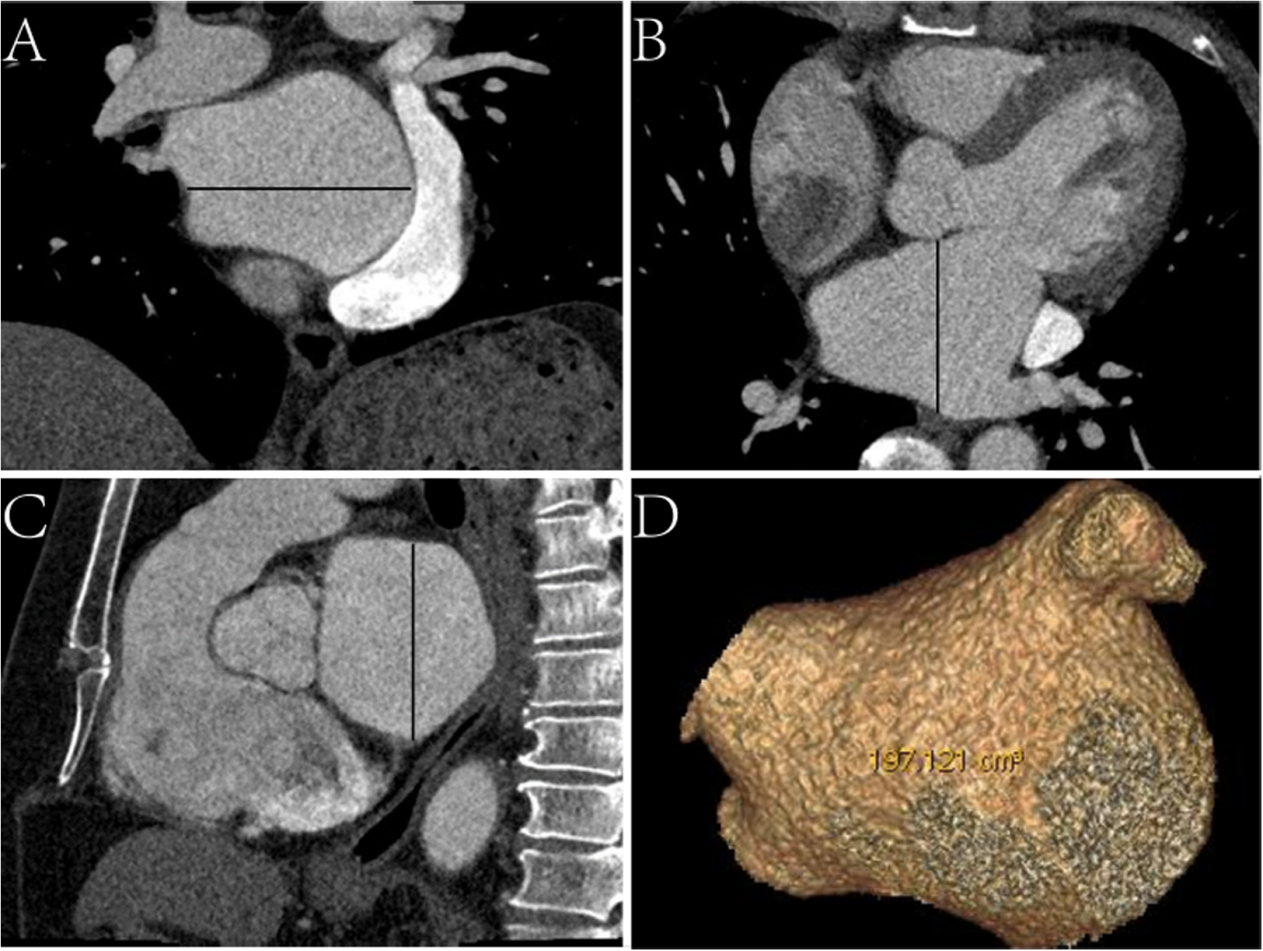
Schematic diagram of measurements of left atrial CT characteristics. **(A)** Coronal CT image, the black line represents the left–right diameter. **(B)** Axial CT image, the black line represents the anteroposterior diameter. **(C)** Sagittal CT image, the black line represents the upper-down diameter. **(D)** Virtual reality CT image displaying the volume.

Epidemiological, laboratory, CT, and echocardiographic data were reviewed. CT image data, including left atrial (LA) characteristics (anteroposterior diameter, left–right diameter, upper-down diameter, and volume) (Figure 1) and EAT characteristics (EAT volume and mean density of the whole heart and LA) were assessed. We used the total area of the EAT to calculate the sum of the EAT volume and then separated it into the whole-heart and periatrial EAT.

### 2.3 Atrial fibrillation ablation

All patients underwent isolated LA PVI. The electrophysiologist decided which type of energy to use [radiofrequency (RF) or cryoablation] based on clinical and imaging factors. Wide-area circumferential RF was exclusively performed in patients with PeAF. The endpoint of the procedure was to isolate the entire pulmonary vein from the LA. After the ablation procedure, if the AF continued, electrical cardioversion was conducted. Before each RF ablation, electroanatomical LA bipolar endocardial voltage mapping was performed using either the EnSite NavX System (St. Jude Medical) or the CARTO 3 system (Biosense Webster Inc.). All the voltage maps with sufficient coverage of the anterior and posterior LA walls were included. Low voltage was defined as <0.5 mV.

### 2.4 CT examination

CT images were acquired using a Revolution CT Scanner (GE Healthcare, Milwaukee, WI, USA) and a SOMATOM Force Scanner (Siemens Medical Solutions, Erlangen, Germany). CT examinations covered the entire heart from 1 cm below the bifurcation of the trachea to the bottom of the heart. The patients were administered a total of 60 mL of ionic contrast material (Ultravist 370, Bayer Schering Pharma) and 40 mL of saline at a rate of 5 mL/s. Two phases of the CT series were obtained 6 s after CT attenuation of the region of interest (ROI) located in the aortic root reached 100 Hounsfield units (HU). The parameters were as follows: tube voltage, 120 kVp; tube current, 400 mA; and reconstructed thickness and increment, 0.75 mm.

### 2.5 Image interpretation

**Figure 2:**
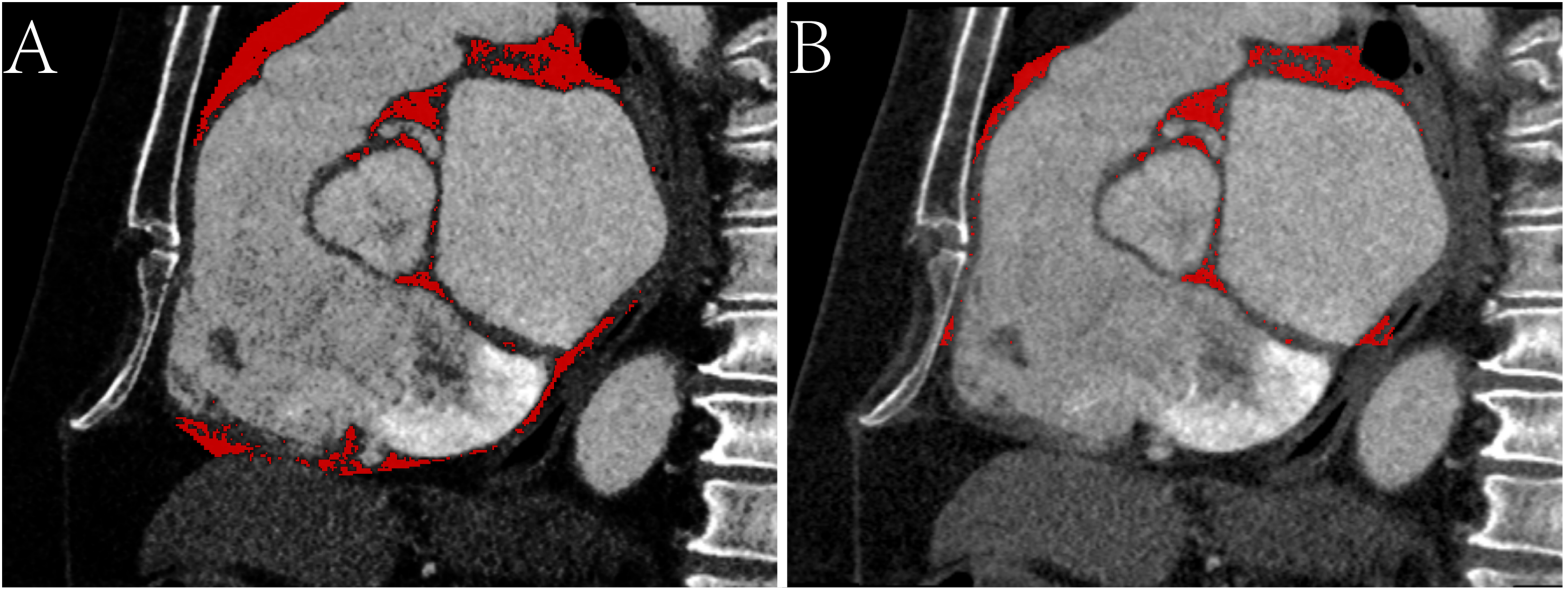
Sagittal CT images of the epicardial adipose tissue on -200 to 0 HU. **(A)** The red area represents the epicardial adipose tissue (EAT) around the whole heart. **(B)** The red area represents the EAT around the atrium.

**Figure 3:**
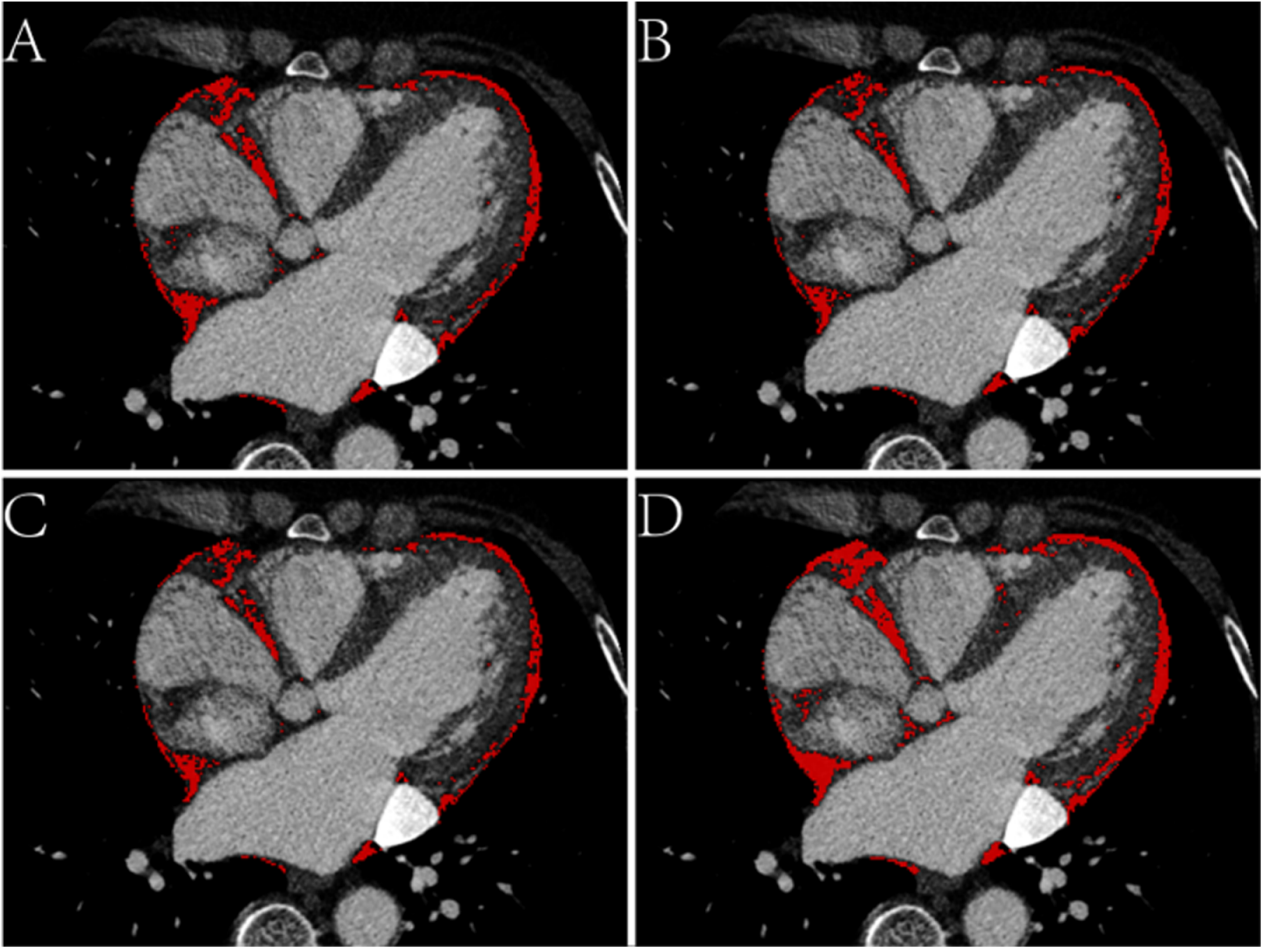
Axial CT images of the epicardial adipose tissue (EAT) based on four attenuation ranges. **(A)** The red area represents the EAT based on -190 to -30 HU. **(B)** The red area represents the EAT based on -195 to -45 HU. **(C)** The red area represents the EAT based on -200 to -45 HU. **(D)** The red area represents the EAT based on -200 to 0 HU.

EAT volume and density were calculated using postprocessing software on a SyngoVia workstation (Cardiac Risk Assessment and Radiomics, MM Research Frontier SyngoVia, VB2.0, Siemens Healthineers, Forchheim, Germany). In total, 16 EAT characteristics of the whole heart and LA were assessed based on four attenuation ranges (−190 to −30 HU, −195 to −45 HU, −200 to −45 HU, −200 to 0 HU) (Figures 2, 3) (11, 12). The EAT features of the whole heart were automatically recognized, while the features of the LA were semiautomatically recognized by outlining the ROI on every 6-mm axial image from the top of the LA appendage to the bottom of the left atrium. After manually delineating and correcting the ROI in each slice, the regions were fused to obtain the desired features. The EAT data around the whole heart in the two measurements were consistent with the automatic recognition software. The Bland–Altman test was used to evaluate the consistency of EAT data only around the atrium and not around the whole heart. Finally, 30 patients were randomly selected by another experienced radiologist to outline the ROI of the EAT around the LA.

### 2.6 Follow-up

A standard 12-lead ECG or systematic 24-h Holter monitor examination was performed at 3 months, 6 months, and 1 year after ablation, or at any time when symptoms were present. AFR was defined as AF, atrial flutter, or atrial tachycardia lasting at least 30 s, recorded 3 months after ablation. Antiarrhythmic drugs were prescribed when AFR was observed 3 months after ablation.

### 2.7 Statistical analysis

The statistical analyses were performed using MATLAB (version 2017, Natick, MA, USA) and SPSS software (version 22.0, IBM Corp., Armonk, NY, USA). The normality of the data was analyzed and the differences between the two groups were evaluated using the chi-square test or Fisher's exact test, or alternatively, the independent *t*-test and Mann–Whitney *U* test. The evaluation of several characteristics in predicting recurrence was conducted by measuring the area under the curve (AUC) of the receiver operating characteristic curve (ROC). A binary logistic regression model (backward Wald) was built to predict recurrence and to screen for independent risk factors. A *P-*value  < 0.1 for the initial screening and a *P-*value < 0.05 for the final screening were considered significant.

## 3 Results

The study included 123 patients who fulfilled the inclusion criteria, of whom 31 (25.2%) had AFR. Tables 1, 2 present an overview of the patients' demographic data and clinical examination characteristics. The recurrence and non-recurrence groups did not vary significantly statistically in terms of sex, smoking history, alcohol intake, history of coronary heart disease, hypertension, hyperlipidemia, hyperuricemia, or stroke. There were no significant differences in brain natriuretic peptide (BNP) and other laboratory indicators between the two groups. The quantitative data of BNP was further analyzed and a *P*-value close to statistical significance was observed between the two groups [754 (363, 1,092) vs. 460 (160, 889), pg/mL, *p* = 0.102].

**Table 1:** Baseline characteristics of the study sample.

Parameter	Non-recurrence (*N* = 92)	Recurrence (*N* = 31)	*P*
Sex			
Male	56	16	0.366
Female	36	15	
Age (years)	60.9 ± 11.2	61.2 ± 12.5	0.918
AF type			
Persistent AF	10	5	
Paroxysmal AF	82	26	0.439
Smoking			0.430
Yes	23	10	
No	69	21	
Alcohol			0.506
Yes	24	10	
No	68	21	
Coronary artery disease			
No	64	22	0.883
Yes	28	9	
Hypertension			0.322
No	51	14	
Yes	41	17	
Dyslipidemia			0.437
No	49	19	
Yes	43	12	
Hyperuricemia			0.502
No	72	26	
Yes	20	5	
Cerebrovascular disease			0.302
No	81	25	
Yes	11	6	

AF, atrial fibrillation.

**Table 2:** Laboratory and ultrasonic characteristics of the study sample.

	Non-recurrence (*N* = 92)	Recurrence (*N* = 31)	*P*
Fibrin (*n* = 119)			0.779
Normal	85	29	
Reduced	4	1	
Creatinine (*n* = 121)			0.877
Normal	84	28	
Reduced	6	3	
BNP (*n* = 78)			0.727
Normal	12	3	
Increased	45	18	
TC (*n* = 118)			0.764
Normal	81	30	
Increased	6	1	
TG (*n* = 118)			0.264
Normal	58	24	
Increased	29	7	
LDL (*n* = 118)			0.471
Normal	86	30	
Increased	1	1	
HDL (*n* = 118)			0.955
Normal	65	23	
Increased	22	8	
CRP (*n* = 65)			0.150
Normal	46	16	
Increased	1	2	
EF (*n* = 121)			0.666
Normal	86	29	
Reduced	4	2	

BNP, brain natriuretic peptide; TC, total cholesterol; TG, triglyceride; LDL, low-density lipoprotein; HDL, high-density lipoprotein; CRP, C-reactive protein; EF, left ventricular ejection fraction in ultrasound examination.

Tables 3, 4 show the correlation between AF phenotype and EAT parameters. The total EAT volume was significantly larger in patients with AFR than in patients without recurrence (*P* < 0.1). The difference was observed in both the entire AF population and the PeAF subgroup population, but not in the paroxysmal AF (PAF) subgroup. In addition, EAT density was assessed in each member of the AF group. Even in the subgroup analysis, there was no evidence of a difference in the average EAT density between those with and without AFR (*P* > 0.1).

**Table 3:** The volume and attenuation of the whole-heart epicardial adipose tissue.

	Total patients			Persistent AF			Paroxysmal AF		
	Non-recurrence	Recurrence	*P*	Non-recurrence	Recurrence	*P*	Non-recurrence	Recurrence	*P*
EAT volume (mL)									
−190 to −30 (HU)	165.9 ± 52.4	194.3 ± 77.7	0.065*	129.5 ± 43.1	187.4 ± 40.3	0.0270*	170.4 ± 51.9	195.7 ± 83.5	0.155
−195 to −45 (HU)	145.59 ± 48.8	171.1 ± 71.5	0.071*	109.7 ± 38.3	162.9 ± 40.0	0.0278*	149.8 ± 48.	172.6 ± 76.6	0.162
−200 to −45 (HU)	145.9 ± 48.9	171.6 ± 71.5	0.071*	110.1 ± 38.4	163.4 ± 39.9	0.0260*	150.3 ± 48.5	173.2 ± 76.6	0.162
−200 to 0 (HU)	207.1 ± 57.5	241.3 ± 87.1	0.048*	172.7 ± 52.2	242.3 ± 36.5	0.0200*	211.3 ± 57.1	241.1 ± 94.3	0.138
EAT attenuation (HU)									
−190 to −30 (HU)	−85.7 (10.7)	−87.9 (11.0)	0.540	−81.3 ± 5.4	−85.2 ± 5.5	0.212	−86.0 (10.3)	−87.3 (11.5)	0.735
−195 to −45 (HU)	−93.0 (11.1)	−95.0 (10.0)	0.610	−90.4 ± 5.4	−93.2 ± 4.8	0.351	−93.5 (11.0)	−95.5 (10.5)	0.801
−200 to −45 (HU)	−93.5 (11.6)	−96.8 (12.0)	0.658	−90. ± 5.4	−93.4 ± 4.6	0.362	−94.1 (11.2)	−96.5 (10.8)	0.846
−200 to 0 (HU)	−71.2 (11.8)	−72.0 (10.0)	0.576	−63.5 (6.7)	−72.0 (14.5)	0.177	−71.9 ± 7.3	−72.3 ± 8.1	0.821

EAT, epicardial adipose tissue; AF, atrial fibrillation.

* *P* < 0.1.

**Table 4:** The volume and attenuation of the periatrial epicardial adipose tissue.

	Total patients			Persistent AF			Paroxysmal AF		
	Non-recurrence	Recurrence	*P*	Non-recurrence	Recurrence	*P*	Non-recurrence	Recurrence	*P*
EAT volume (mL)									
−190 to −30 (HU)	93.3 ± 32.4	103.8 ± 39.2	0.185	73.8 ± 21.3	104.8 ± 33.8	0.047*	95.7 ± 32.8	103.6 ± 40.7	0.374
−195 to −45 (HU)	80.2 ± 29.6	89.7 ± 35.1	0.141	61.5 ± 18.2	89.3 ± 31.8	0.048*	82.5 ± 29.9	89.9 ± 36.2	0.302
−200 to −45 (HU)	80.5 ± 29.5	90.1 ± 35.1	0.141	61.7 ± 18.3	89.6 ± 31.8	0.048*	82.8 ± 29.9	90.2 ± 36.3	0.302
−200 to 0 (HU)	118.7 ± 36.7	132.3 ± 46.1	0.142	100.6 ± 26.7	139.3 ± 34.2	0.031*	120.9 ± 37.3	130.9 ± 48.4	0.338
EAT attenuation (HU)									
−190 to −30 (HU)	−84.0 (10.8)	−85.0 (13.0)	0.600	−80.0 ± 5.4	−81.8 ± 6.8	0.587	−84.0 (11.0)	−85.5 (11.0)	0.637
−195 to −45 (HU)	−92.0 (11.0)	−94.0 (12.0)	0.550	−90.1 ± 5.7	−90.8 ± 6.4	0.832	−92.5 (12.0)	−94.9 (12.0)	0.625
−200 to −45 (HU)	−93.0 (11.0)	−94.0 (13.0)	0.675	−63.1 ± 4.7	−65.6 ± 10.5	0.528	−93.9 (11.0)	−94.5 (12.0)	0.674
−200 to 0 (HU)	−69.0 (11.0)	−70.0 (13.0)	0.564	−88.1 ± 5.8	−90.6 ± 6.2	0.456	−69.2 ± 7.4	−70.1 ± 8.3	0.607

EAT, epicardial adipose tissue.

* *P* < 0.1.

When the periatrial EAT was examined, patients with PeAF in the recurrence group had a significantly higher periatrial EAT volume than those without such a recurrence (*P* < 0.05). Nevertheless, neither the whole group nor the patients with paroxysmal AF showed this variance (*P* > 0.1). There was also no statistically significant difference in the density of atrial epicardial adipose tissue between the groups with AFR and without recurrence, in both the whole population and subgroup analyses.

LA volume, LA anteroposterior diameter, and LA left–right diameter were all greater in the AFR group (*P* < 0.05). In contrast, neither the diameter nor the volume of the LA varied significantly among the PeAF subgroup (*P* > 0.1). The LA upper-down diameter did not differ significantly between individuals with and without AFR (*P* > 0.1) (Table 5).

**Table 5:** CT characteristics of the left atrium in the study sample.

	Total patients			Persistent AF			Paroxysmal AF		
	Non-recurrence	Recurrence	*P*	Non-recurrence	Recurrence	*P*	Non-recurrence	Recurrence	*P*
Anteroposterior diameter (mm)	43.7 ± 8.0	47.4 ± 7.4	0.021*	46.8 ± 5.7	50.4 ± 8.4	0.344	43.3 ± 12.3	46.9 ± 7.2	0.045*
Left–right diameter (mm)	74.7 ± 9.1	78.6 ± 7.9	0.037*	80.4 ± 8.5	83.2 ± 4.7	0.503	73.2 (9.5)	78.5 (8.2)	0.035*
Upper-down diameter (mm)	64.8 (10.9)	65.1 (15.4)	0.149	154.5 (49.8)	164.3 (48.4)	0.713	62.8 (10.6)	65.1 (15.8)	0.133
Volume (mL)	127.0 (48.3)	145.9 (34.6)	0.015*	70.5 (11.9)	69.1 (18.2)	0.391	125.3 (46.7)	144.2 (36.5)	0.029*

* *P* < 0.1.

**Figure 4:**
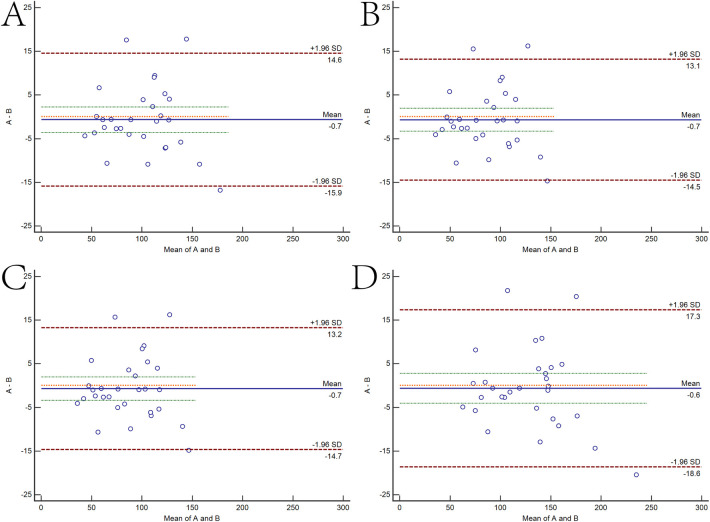
Bland–Altman scatter plot of four attention range setting methods for two surveyors. **(A)** epicardial adipose tissue (EAT) as measured by two surveyors based on -190 to -30 HU. **(B)** EAT as measured by two surveyors based on -195 to -45 HU. **(C)** EAT as measured by two surveyors based on -200 to -45 HU. **(D)** EAT as measured by two surveyors based on -200 to 0 HU.

**Figure 5:**
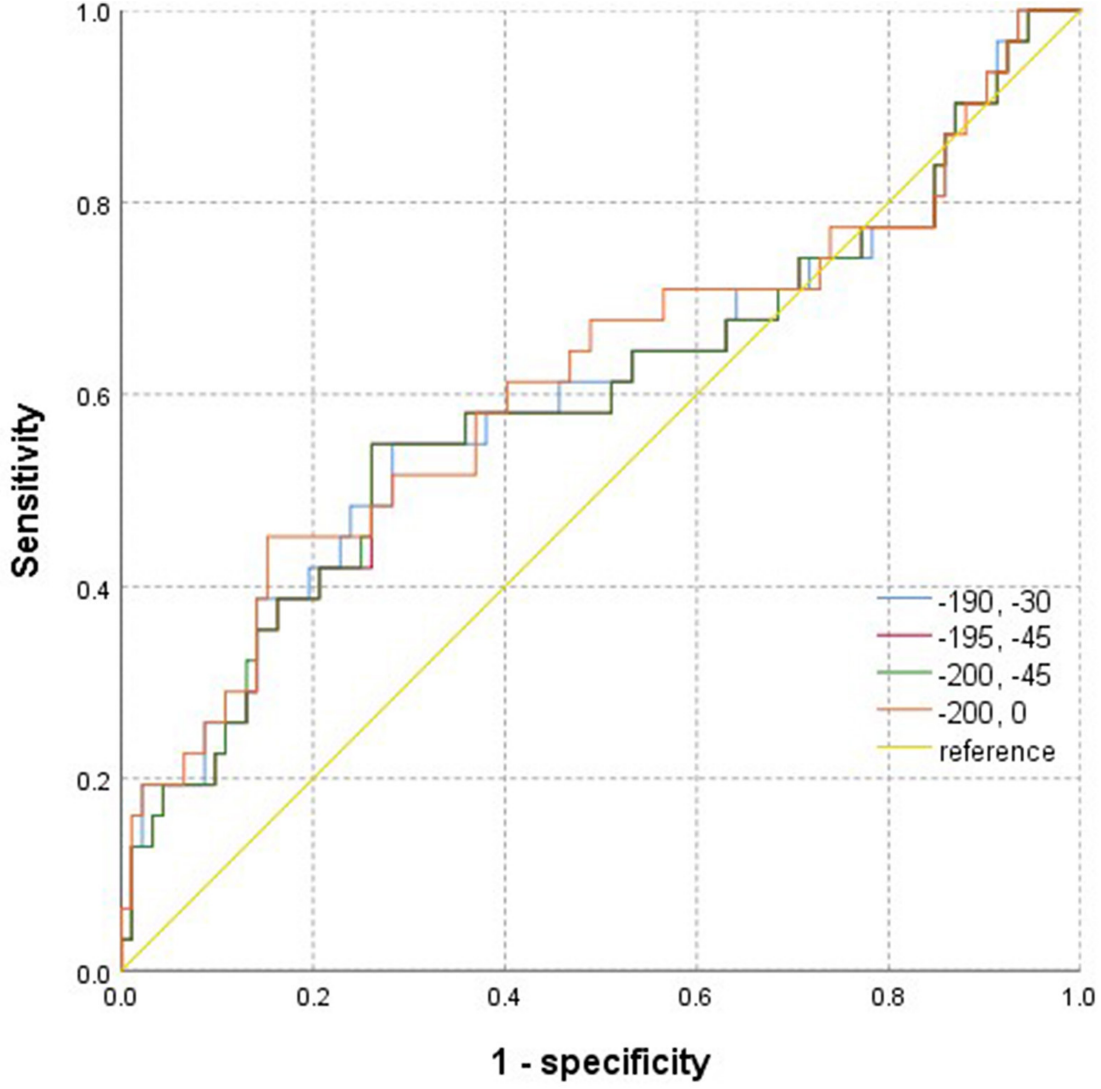
ROC images of epicardial adipose tissue volume measured based on four attenuation ranges for predicting the recurrence of atrial fibrillation after radiofrequency ablation.

The two independent readers exhibited a high degree of consistency in the four thresholds around the heart chamber (Figure 4), and the data tendencies were consistent for each of the four different fat thresholds. The AUC of the −200 to 0 HU threshold for whole-heart fat was 0.615. The other three threshold values for predicting AFR were lower with 0.601 for −190 to −30 HU, 0.595 for −195 to −45 HU, and 0.596 for −200 to −45 HU. As shown in Figure 5, the regression analysis accounted for the maximum AUC of the whole-heart EAT (−200 to 0 HU). The most widely used whole-heart EAT criterion (−190 to −30 HU) was also included as a significant variable in the regression model. The regression analysis showed that the whole-heart EAT volume and left atrial anteroposterior diameter were independent risk factors for AFR (*P* < 0.05) (Table 6).

**Table 6:** Multivariate logistic regression model for predicting atrial fibrillation recurrence after radiofrequency ablation.

Variables	*β* value	OR value (95% CI)	*P*-value
The total EAT volume (mL) [−200 to 0 (HU)]	0.01	1.01 (1.00–1.01)	0.035*
Anteroposterior diameter	0.06	1.06 (1.00–1.12)	0.045*
Constant	−5.175	0.01	0.001*

* *P* < 0.05.

## 4 Discussion

Approximately 30% to 40% of individuals have AFR after the first ablation treatment (5). Previous studies have investigated the correlation between CT features of EAT and AFR, however, the results have been inconsistent (8–10, 13). The results of this study indicate that the EAT volume and LA anteroposterior diameter are of great significance in predicting AFR.

Our study assessed that the AFR was 25.2%, which is consistent with a previously documented occurrence rate (5). Patients diagnosed with AFR had a higher probability of having a greater LA diameter and volume compared to those without recurrence. This discovery strongly corroborates the conclusions of previous research that establish a connection between echocardiographic measurements, such as the diameter and volume of the LA and the risk of arrhythmia recurring following catheter ablation (14). Hof et al. discovered that with each 10 mL increase in LA volume, there was a 1.14 adjusted odds ratio (OR) for AFR (15). A meta-analysis of 11 studies found a positive link between an elevated average LA volume and an increased incidence of recurrence (16). This research revealed that the anteroposterior diameter of the LA was a significant factor that influenced AFR. Studies (17) have indicated that BNP might be a biomarker for predicting AFR, but no difference was observed in our study, which may be due to incomplete data.

Inconsistent data exist regarding the function of the EAT and its effect on AFR following ablation. The EAT volume corrected by body surface area was considered an independent factor affecting AFR (18). Al Chekakie et al. found that patients with PeAF had a greater volume of pericardial fat than those with PAF or sinus rhythm (19). EAT volume was associated with the risk of AFR following PVI ablation, according to previous research (8, 20). Other studies have failed to establish a statistically significant correlation between EAT and AFR following ablation (13). In support of the previous conclusion, our research emphasizes the significance of EAT volume in predicting AFR after ablation. In contrast to the PAF group, this distinction was more pronounced in the PeAF group. The difference in the overall volume of EAT was more pronounced compared to the difference in the volume of periatrial EAT. Compared to a previous study (21), four different distinct fat thresholds were analyzed in our study. Although the data trends exhibited consistency, the difference was more pronounced in the −200 to 0 HU range compared to the other thresholds.

The prognosis of patients with recurrent episodes of AF may be enhanced by assessing EAT as a marker of ablation efficiency. Nevertheless, information on the distribution of EAT around the LA and its predictive implications in patients undergoing AF catheter ablation remains limited. Nakanishi et al. first demonstrated that the periatrial EAT volume estimated using multidetector CT accurately predicted the development of new-onset AF in patients with coronary artery disease (22). Tsao et al. discovered that the EAT surrounding the LA had a significant impact on the success of the ablation procedure. A review also emphasized the predictive role of peri-LA EAT in AFR after ablation (21). However, there was no difference in the volume of EAT around the LA between patients with PAF and PeAF (9). In our study, one interesting finding was that the periatrial EAT volume was much greater in patients with PeAF who had recurrence compared to those who did not. Contrary to what was expected, our research did not find a significant difference between those who had a recurrence of PAF and those who did not. This conclusion agrees with that of Huber et al., who discovered that LA-enhancing EAT was independently linked to recurrence after AF ablation (23). Shin and associates focused on the EAT and AF to find inflammatory indicators. Every patient in their experiment had measurements of their periventricular, periatrial, and total EAT volumes. They discovered that periatrial fat in patients with PeAF was thicker than in patients with PAF (24). Our findings emphasize that higher periatrial EAT volume was associated with the recurrence of PeAF after ablation, but the general rather than local effects of EAT play a role in the onset of early recurrence of AF after ablation. Compared to PAF, the pathogenesis of PeAF seems to be more linked with atrial metabolism, although confounding factors may be involved.

The role of EAT density in the early recurrence of AF remains unknown. Situated between the visceral pericardium and the myocardium, EAT is a unique kind of visceral adipose tissue (25). Significant paracrine and vasocrine effects are exerted on nearby cardiomyocytes by the EAT's advantageous position (26). The production of fatty acids and proinflammatory adipokines is one way that the EAT changes from an anti-inflammatory to a pro-inflammatory phenotype in disease situations. EAT radiodensity can reflect the pathological status. Measured by adipose tissue attenuation on CT, periatrial inflammation was associated with AF independent of LA size (27). Inflammation causes higher attenuation of adipose tissue, as assessed by CT in patients with recurrence of AF after catheter ablation (28). It has also been reported that EAT radiodensity is associated with the occurrence, severity, and recurrence of AF (29, 30). Our investigation did not reveal any indication of a difference in average EAT density between patients with and without AFR, which is in contrast to previous results. Similar to previous research, our analysis demonstrated that the attenuation of adipose tissue posterior to the LA was not a predictor when confounding variables were taken into account (30). A possible explanation for this result may be that inflammation may be a factor in the recurrence of AF. However, inflammation alone is not sufficient to determine recurrence. T cells may also be linked to the influence of EAT on AFR (2).

This study suggests that EAT may play a role in risk stratification to identify patients who are more likely to develop progressive and recurrent AF after catheter ablation. The link between EAT and AF might be explained by a number of pathophysiological processes. Cardiovascular disease is associated with the EAT, which is the most metabolically active adipose depot next to the heart (31). As opposed to pericardial fat, EAT has particular properties such as sharing the same blood circulation as the myocardium and exhibiting cell-to-cell contact or infiltration. Furthermore, a few studies have shown a correlation between total adiposity and shorter effective refractory durations in the pulmonary veins in obese individuals with AF (32). This resulted in the hypothesis that individuals with high levels of obesity would be more susceptible to the onset of AF. However, during AF ablation treatments, a previous research study created 3D electroanatomical maps and discovered that regions of fragmented signals were close to the EAT (33). This discovery implies that AF might be triggered by EAT.

This research has some limitations. First, it was constrained by the characteristics of observational research conducted at a single center. However, a multivariate analysis was carried out in order to exclude the influence of important identified factors on the study results. Second, EAT features on echocardiography were not included in the study, mainly because echocardiography is an operation highly dependent on operator proficiency, especially in EAT measurement. The last limitation is a lack of studies on the mechanisms behind the correlation between pericardial or EAT volume and AFR. More research is needed to understand the potential mechanisms behind the association between AFR and the amount of fat in the pericardium or epicardium. Further studies with larger sample sizes and a multicenter methodology should be explored to enhance the applicability of the results.

## 5 Conclusion

In conclusion, the EAT was found to play a significant role in AFR, and the effect was more pronounced in patients with PeAF than in those with PAF. The influence of total EAT volume was higher than that of the periatrial EAT volume. The data trends obtained for the four fat thresholds were consistent.

## Data Availability

The datasets presented in this study can be found in online repositories. The names of the repository/repositories and accession number(s) can be found in the article/Supplementary Material.
